# Characterization of the global transcriptome for *Pyropia haitanensis* (Bangiales, Rhodophyta) and development of cSSR markers

**DOI:** 10.1186/1471-2164-14-107

**Published:** 2013-02-16

**Authors:** Chaotian Xie, Bing Li, Yan Xu, Dehua Ji, Changsheng Chen

**Affiliations:** 1 Fisheries College, Jimei University, Xiamen, Fujian Province 361021, People's Republic of China

**Keywords:** *Pyropia haitanensis*, Transcriptome, Carbon fixation pathway, cSSR markers

## Abstract

**Background:**

*Pyropia haitanensis* is an economically important mariculture crop in China and is also valuable in life science research. However, the lack of genetic information of this organism hinders the understanding of the molecular mechanisms of specific traits. Thus, high-throughput sequencing is needed to generate a number of transcriptome sequences to be used for gene discovery and molecular marker development.

**Results:**

In this study, high-throughput sequencing was used to analyze the global transcriptome of *P. haitanensis*. Approximately 103 million 90 bp paired-end reads were generated using an Illumina HiSeq 2000. *De novo* assembly with paired-end information yielded 24,575 unigenes with an average length of 645 bp. Based on sequence similarity searches with known proteins, a total of 16,377 (66.64%) genes were identified. Of these annotated unigenes, 5,471 and 9,168 unigenes were assigned to gene ontology and clusters of orthologous groups, respectively. Searching against the KEGG database indicated that 12,167 (49.51%) unigenes mapped to 124 KEGG pathways. Among the carbon fixation pathways, almost all the essential genes related to the C3- and C4-pathways for *P. haitanensis* were discovered. Significantly different expression levels of three key genes (Rubisco, PEPC and PEPCK) in different lifecycle stages of *P. haitanensis* indicated that the carbon fixation pathway in the conchocelis and thallus were different, and the C4-like pathway might play important roles in the conchocelis stage. In addition, 2,727 cSSRs loci were identified in the unigenes. Among them, trinucleotide SSRs were the dominant repeat motif (87.17%, 2,377) and GCC/CCG motifs were the most common repeats (60.07%, 1,638). High quality primers to 824 loci were designed and 100 primer pairs were randomly evaluated in six strains of *P. haitanensis*. Eighty-seven primer pairs successfully yielded amplicons.

**Conclusion:**

This study generated a large number of putative *P. haitanensis* transcript sequences, which can be used for novel gene discovery and gene expression profiling analyses under different physiological conditions. A number of the cSSR markers identified can be used for molecular markers and will facilitate marker assisted selection in *P. haitanensis* breeding. These sequences and markers will provide valuable resources for further *P. haitanensis* studies.

## Background

*Pyropia*, a genus of marine red algae, is one of the most economically important mariculture crops. It has an annual harvest of more than 120,000 t (dry weight) and a value of over US $2 billion per year [1-3]. With the expansion of artificial seeding and the development of the floating culture method, farming and processing of *Pyropia* has now become one of the largest seaweed industries in East Asian countries, including China, Japan and South Korea [3]. In China, two major cultivars, *Pyropia yezoensis* Ueda and *Pyropia haitanensis* Chang et Zheng, are distributed in northern China and southern China, respectively. *P. haitanensis*, a typical warm temperate zone species originally found in Fujian Province, comprises 75% of the total production of cultivated *Pyropia* in China [4,5].

*Pyropia* are not only economically important, but also have high basic research value. As sessile organisms that inhabit the intertidal zone, an environment of rapidly changing physical conditions due to the turning tides, *Pyropia* have high levels of tolerance to various abiotic stressors such as desiccation, osmotic shock, temperature, and light [1]. Furthermore, *Pyropia* differ from most terrestrial plants in many aspects of their biology, such as their unique heteromorphic digenetic life cycle, their special pathway for carbon assimilation in photosynthesis, their genetic chimera of blades, their ability to accumulate iodine, their original composition of their cell walls, and their associated cell wall synthesis pathways [3]. These specific characteristics present opportunities for new discoveries in *Pyropia*. Recently, *P. yezoensis* has been recognized as a useful model organism for fundamental and applied studies of marine algae [3,6], and a great deal of studies have been conducted to understand the special characteristics of *Pyropia*[1,7-12]. A whole genome sequencing project is also ongoing. However, for *P. haitanensis*, which has more primitive properties and special biological processes than *P. yezoensis*[13,14], limited studies have been conducted, and many aspects of its biology remain poorly explored. Currently, only 6035 ESTs and 140 nucleotide sequences are deposited in NCBI database for *P. haitanensis*, and its mitochondrial genome was just been sequenced [14]. The whole genome of *P. haitanensis* may not be sequenced for years. The limited genomic sequence resources have hampered studies to elucidate the molecular mechanisms of specific traits and understand the complex mechanisms of stress tolerance in *P. haitanensis*.

Transcriptome analysis is an attractive alternative to whole genome sequencing. A transcriptome is a complete set of transcripts in a cell or an organism at a specific developmental stage or under a physiological condition [15-17]. The transcriptome can provide useful information about gene expression, gene regulation, and amino acid content of proteins. Therefore, transcriptome analysis is essential to interpret the functional elements of the genome and reveal the molecular constituents of cells and tissues [16,17]. During the last few years, various technologies have been used to study the *Pyropia* transcriptomes, including EST sequencing [7,8,13], and microarrays [9]. However, microarrays are limited by background and cross hybridization problems and only measure the relative abundance of transcripts. Moreover, only predefined sequences are detected. Traditional sequencing methods for the generation of ESTs require costly and time-consuming approaches involving cDNA library construction, cloning, and labor intensive Sanger sequencing. These problems have limited the transcriptome analysis in *Pyropia*.

This situation has changed with the introduction of Next Generation Sequencing (NGS) technology. NGS technology, including the Illumina HiSeq 2000, the Roche/454 Genome Sequencer FLX Instrument and the ABI SOLiD System, is considered a powerful and cost-efficient tool for advanced research in many areas, including re-sequencing, microRNA expression profiling, DNA methylation, and especially *de novo* transcriptome sequencing for non-model organisms [18]. These NGS platforms can sequence in parallel massive amounts of DNA molecules derived directly from mRNA, producing millions or even billions of high-quality short reads. Previous studies have confirmed that the relatively short reads can be effectively assembled, especially with the great advantage of paired-end sequencing [15-18]. The Illumina transcriptome or whole genome *de novo* sequencing and assembly have been successfully used for several non-model organisms [17,19-21]. Furthermore, NGS has also significantly accelerated and improved the sensitivity of gene-expression profiling, and is expected to boost collaborative and comparative genomics studies [19]. Nevertheless, despite its obvious advantages, NGS was only recently first undertaken in *Pyropia* to investigate the transcriptome of *P. yezoensis*[11].

Thus, the present study aimed to characterize *P. haitanensis*’ functional genome and identify novel genes. The high-throughput sequencing platform Illumina HiSeq 2000 was used to profile the transcriptome of *P. haitanensis*. We constructed a library with mixed samples, including sporophytes and gametophytes of *P. haitanensis*, which were cultured under different conditions. Nearly 120 million reads totaling over 9 billion bp of high-quality DNA sequence with an average read length of 90 bp were obtained. These sequences were assembled into 24,575 unigenes by trinity *de novo* transcriptome assembly software [20], and approximately 70% of these unigenes were already annotated, as identified by BLAST searches against the SwissProt (http://expasy.org/tools/blast), Kyoto Encyclopedia of Genes and Genomes (KEGG, http://www.genome.jp/kegg), Clusters of orthologous groups (COG, http://www.ncbi.nlm.nih.gov/COG/), Nr (http://blast.ncbi.nlm.nih.gov/Blast.cgi) and gene ontology (GO) databases. A total of 2,727 cSSR (cDNA simple sequence repeat) markers of *P. haitanensis* also have been developed based on these unigenes by MicroSAtellite (MISA, http://pgrc.ipk-gatersleben.de/misa/) software. These assembled, annotated transcriptome sequences and SSR markers provide a valuable genomic resource for further studying the molecular basis of *P. haitanensis*’ special biological features and for marker-assisted selective breeding in *P. haitanensis*.

## Results

### Illumina sequencing and *de novo* assembly

To characterize the functional genome and identify novel genes in *P. haitanensis*, we constructed a Solexa cDNA library with mixed samples as listed in Additional file 1. Using Illumina paired-end sequencing technology, each reaction can yield 2×90 bp independent reads from either end of a DNA fragment. In this study, a total of 119,718,486 reads from the library were obtained. After removing adaptor sequences, empty reads and low quality sequences, 102,967,578 (86.01%) clean reads were obtained with 96.43% Q20 bases (base quality greater than 20) (Table 1). Based on the high quality reads, a total of 44,269 contigs from the library were assembled with an average length of 375 bp by using the *de novo* assembly program Trinity.

**Table 1:** **Summary of the *P. haitanensis *transcriptome**

**Item**	**Number**
Total number of raw reads	119,718,486
Total number of clean reads	102,967,578
Total base pairs (bp)	9,267,082,020
Average read length	90
Q20	96.43%
GC percentage	63.99%
Total number of contigs	44,269
Mean length of contigs	375
Total number of unigenes	24,575
Mean length of unigenes	645
N50	913

**Figure 1:**
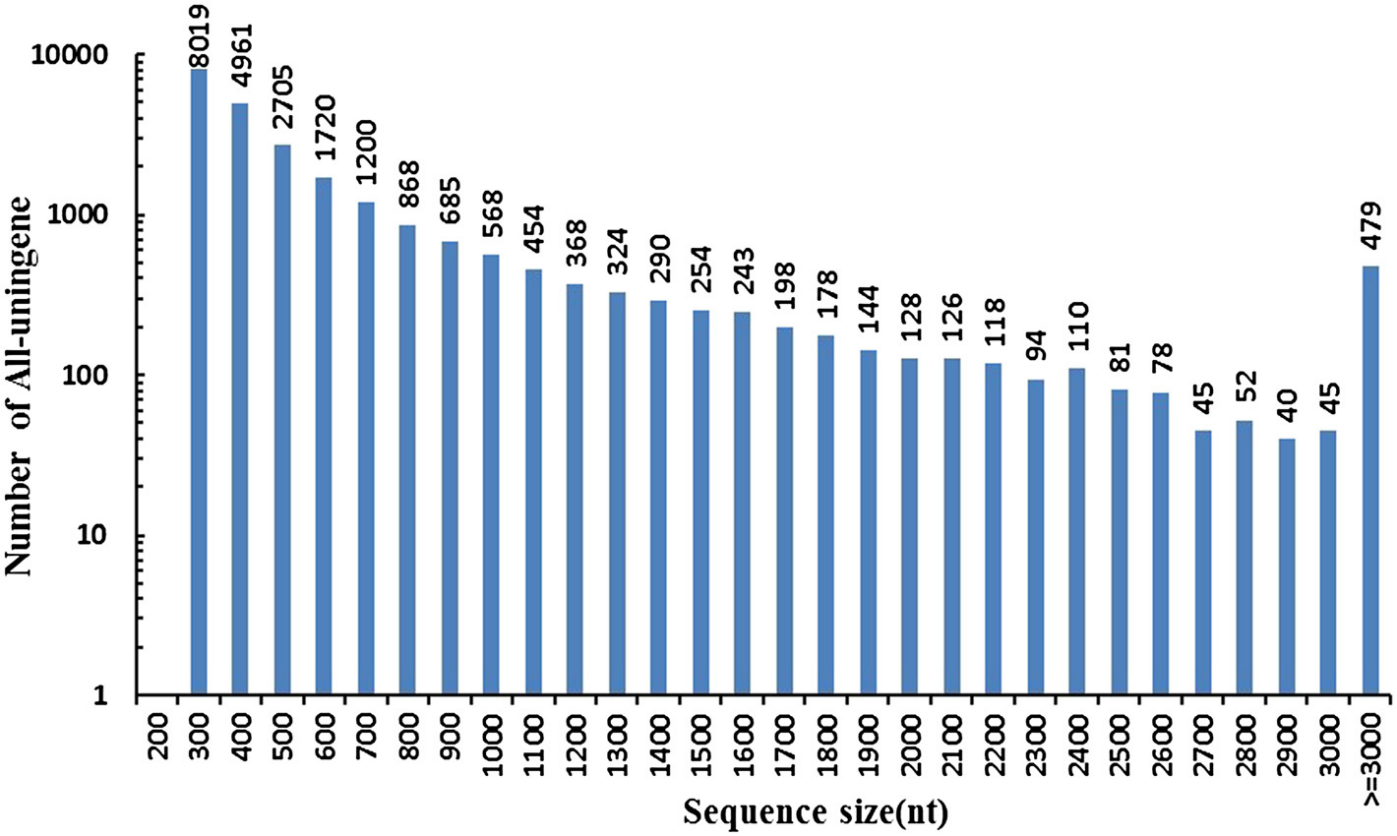
**Length distribution of all unigenes mapped from contigs of the *P. haitanensis *transcriptome.**

With paired-end reads, it is possible to identify contigs derived from the same transcript as well as the distances between these contigs. We, therefore, mapped the reads back to the contigs, and then with the paired-end information joined contigs into unigenes whose sequences could not be extended on either end. As a result, 24,575 unigenes with an average length of 645 bp were obtained. The length of these assembled unigenes ranged from 200 to 11,338 bp, their size distribution is shown in Figure 1. In these unigenes, the GC percentage was 63.99%. To demonstrate the quality of sequencing data, we randomly selected 10 unigenes and designed 10 pairs of primers for RT-PCR amplification. In this analysis, all 10 primer pairs resulted in a band of the expected size, and the identity of all ten PCR products were confirmed by Sanger sequencing (data not shown).

### Functional annotation and classification

For validation and annotation of assembled unigenes, sequence similarity searches were conducted against the NCBI non-redundant protein (Nr) database and the Swiss-Prot protein database using BLASTx algorithm with an E-value threshold of 10^-5^. The results indicated that 15,535 (63.21%) of 24,575 unigenes showed significant similarities to known proteins in the Nr database (Additional file 2) and 12,226 (49.75%) had BLAST hits in the Swiss-Prot database (Additional file 2). Furthermore, to estimate the number of annotated unigenes that matched to unique genes in the two databases, the two files were filtered for duplicate protein accessions and 16,377 (66.64%) annotated unigenes were obtained. Because of the lack of genomic information in *Pyropia*, the remaining 8,198 (33.36%) unigenes could not be matched to any known genes. These annotated unigenes formed a potential pool for gene identification in *Pyropia*.

GO is an international standardized gene functional classification system that offers a dynamic, updated, and controlled vocabulary and strictly defined concepts to comprehensively describe the properties of genes and their products in any organism. In this study, on the basis of Nr annotation, the Blast2GO program [22] was used to obtain GO annotation for unigenes annotated by Nr. Then the WEGO software [23] was used to perform a GO functional classification for these unigenes. In total, 5,471 unigenes with BLAST matches to known proteins were assigned to gene ontology classes with 30,446 functional terms (Additional file 2, Figure 2). Assignments to the cellular component category made up the majority (14,093; 46.29%), followed by biological processes (10,274; 33.74%) and molecular functions (6,079; 19.97%).

**Figure 2:**
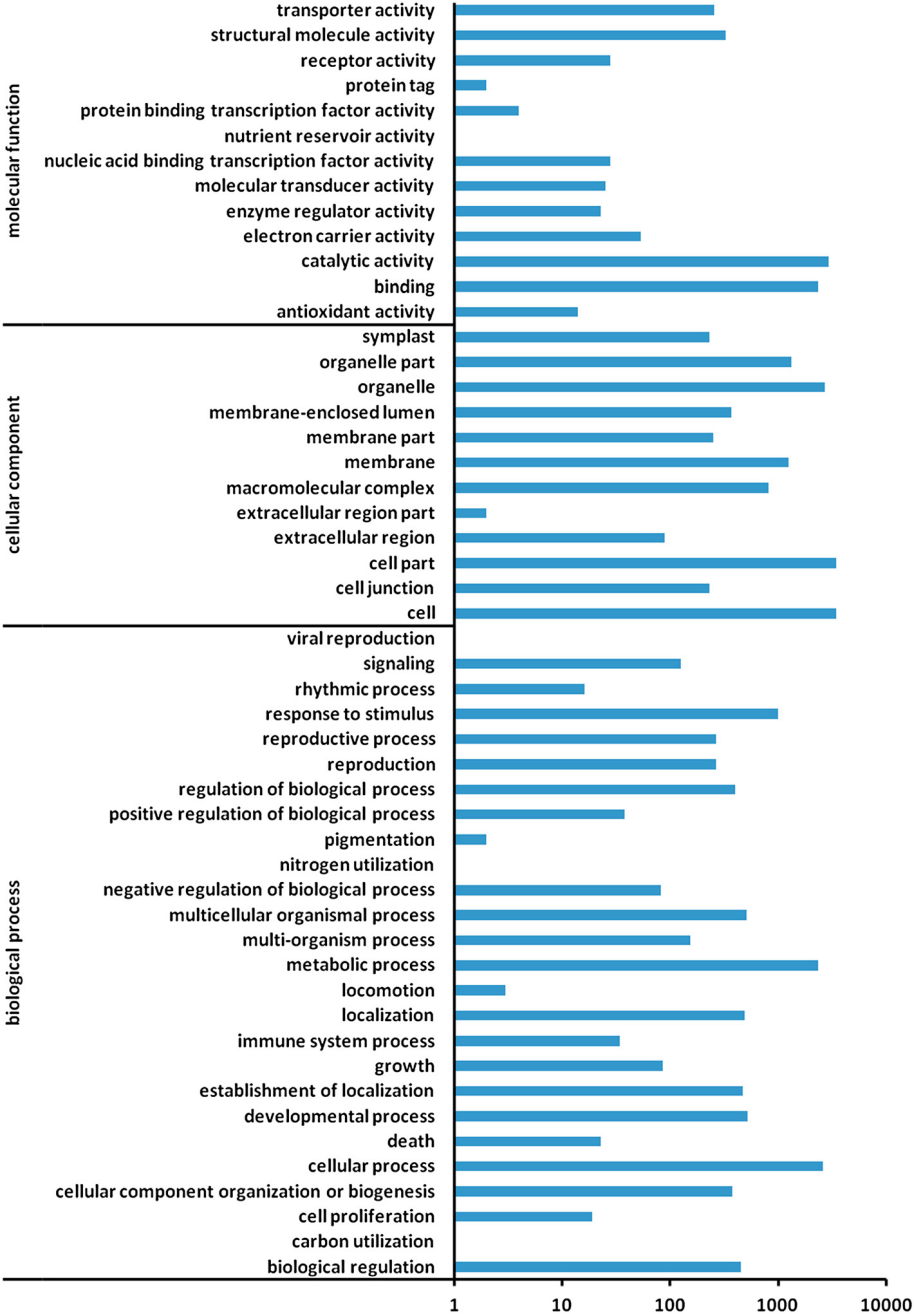
**Gene Ontology (GO) classification of assembled unigenes mapped from contigs of the *P. haitanensis *transcriptome.**

The assigned functions of the unigenes covered a broad range of GO categories. Under the cellular component, the cell, cellular parts and organelles represented the majority of this category (Figure 2). Under the biological process category, metabolic processes and cellular processes were prominently represented (Figure 2). It is noteworthy that 992 unigenes are involved in responses to stimuli (Figure 2, Additional file 2). For the molecular function category, catalytic activity and binding were the major classifications (Figure 2).

**Figure 3:**
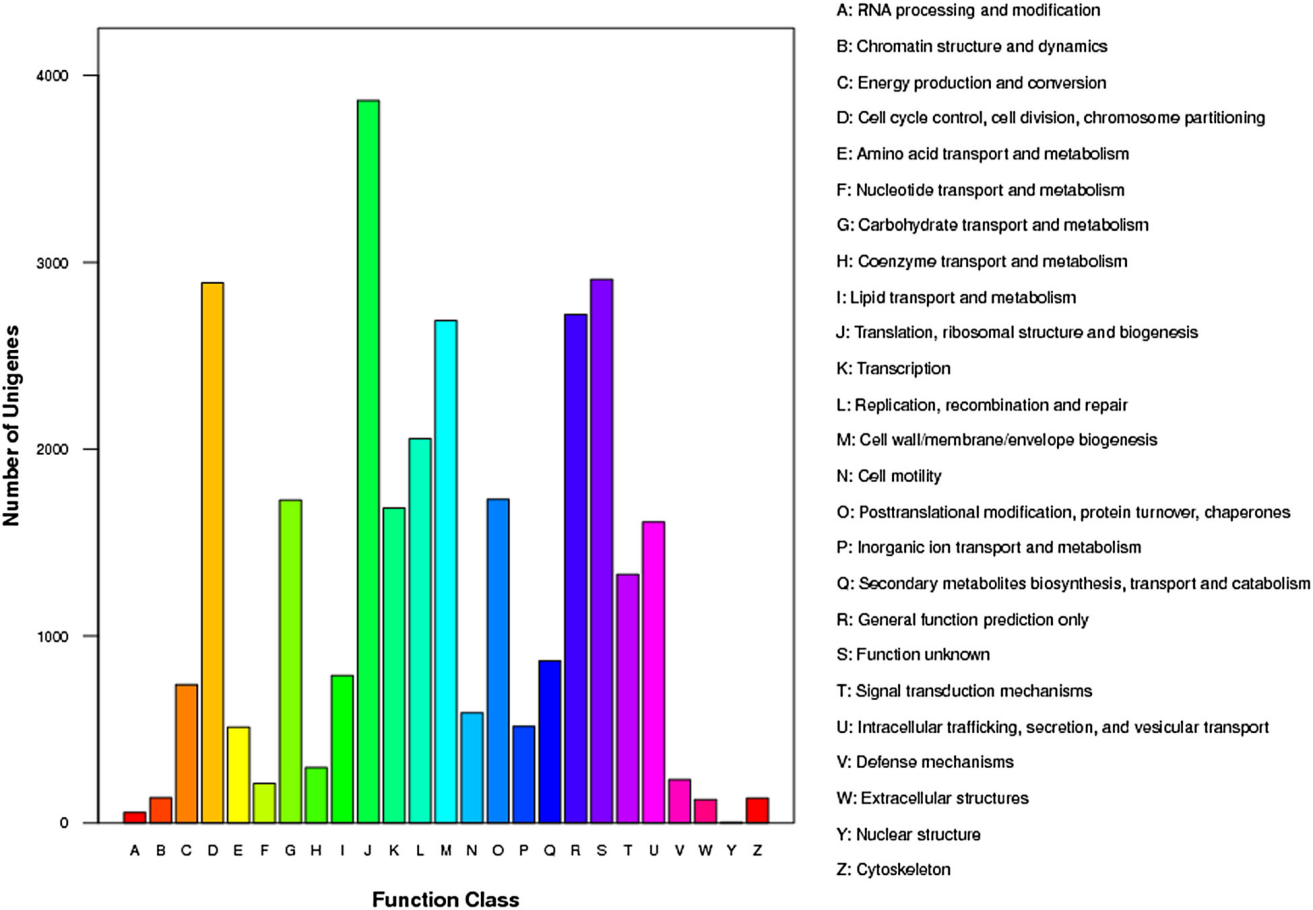
**Histogram presentation of clusters of orthologous groups (COG) classification of assembled unigenes mapped from contigs of the *P. haitanensis *transcriptome.**

To further evaluate the completeness of our transcriptome library and the effectiveness of our annotation process, all annotated unigenes were aligned to the COG database to predict and classify possible functions. A total of 9,168 sequences of 15,535 Nr hits were assigned to the COG classifications (Additional file 2, Figure 3). Among the 25 COG categories, the clusters for translation, ribosomal structure and biogenesis (3,866, 12.71%) represented the largest groups, followed by cell cycle control, cell division, and chromosome partitioning (2,892, 9.51%), general function (2,722, 8.95%), and cell wall/membrane/envelope biogenesis (2,689, 8.84%). Only three unigenes were assigned to nuclear structure (Figure 3). In addition, 232 unigenes were found to be involved in “defense mechanisms”.

The KEGG Pathway database records the networks of molecular interactions in the cell and variants of them specific to particular organisms. Pathway-based analyses can help to further understand the biological functions and interactions of genes. Based on a comparison against the KEGG database using BLASTx with an E-value cutoff of <10^-5^, 12,167 (49.51%) of the 24,575 unigenes had significant matches in the database and were assigned to 124 KEGG pathways (Additional file 2). The pathways represented the most by the unigenes were the metabolic pathway (4,259 unigenes), RNA transport (3,759 unigenes), and the mRNA surveillance pathway (3,375 unigenes).

### Pathway of carbon fixation in *P. haitanensis*

**Figure 4:**
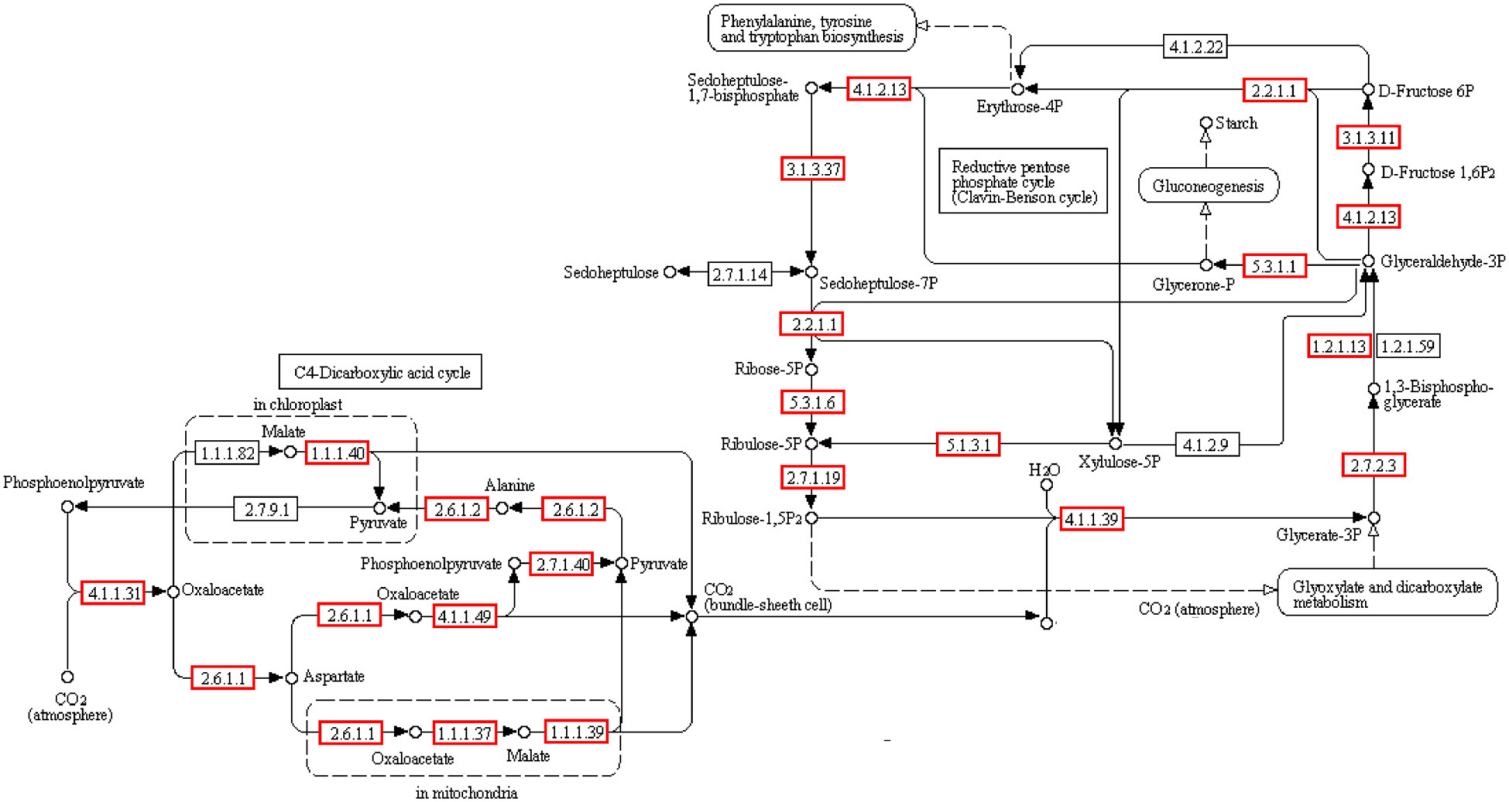
**Putative pathway of carbon fixation in *P. haitanensis *generated by KEGG. **The numbers within the small boxes are enzyme codes. The small boxes in red are enzymes identified in this study. The small boxes in black are enzymes not identified in this study.

**Figure 5:**
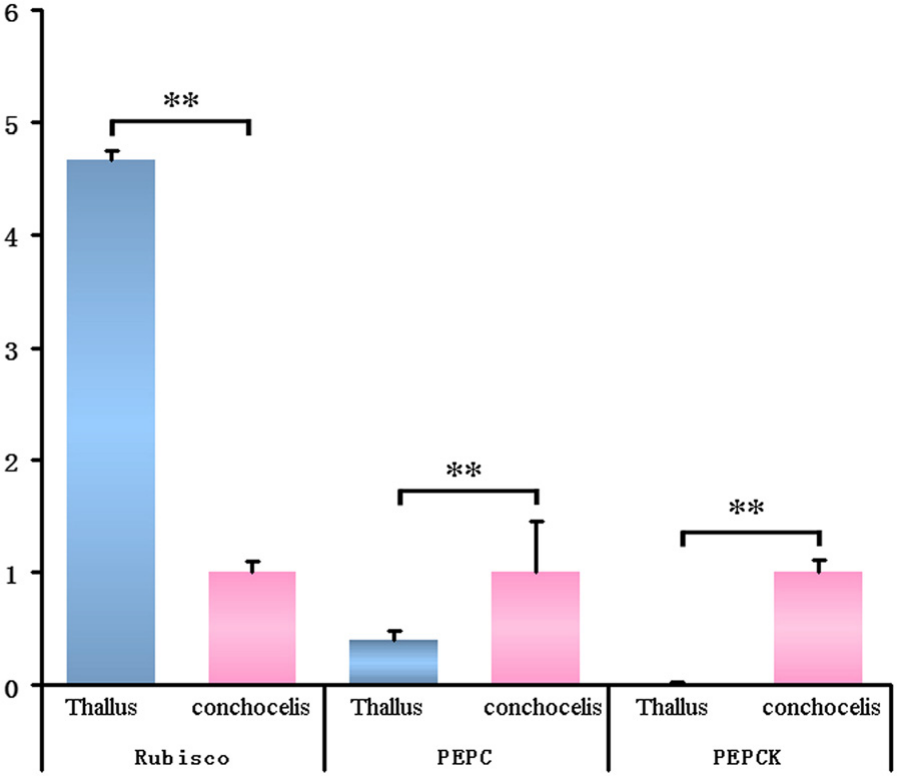
**The relative expression levels of PEPC, PEPCK and Rubisco in the conchocelis and thallus of *P. haitanensis*.** ** *P*<0.01.

Carbon fixation is the most important biological process in all photosynthetic organisms. By blasting the KEGG database, we identified most of the key genes related to the C3 pathway (49 unigenes) and the C4 pathway (24 unigenes) of carbon fixation in *P. haitanensis*, except for EC 4.1.2.9, EC 4.1.2.22, EC 1.1.1.82 and EC 2.7.9.1 (Figure 4, Table 2). Several studies have reported that the carbon fixation mechanisms in *Pyropia* are different between free-living conchocelis and thallus and that a special C4-like carbon-fixation pathway might exist in the sporophytes [13,24,25]. However, there has been no direct evidence so far to support this. Since the key enzymes involved in the C4 pathway are phosphoenolpyruvate carboxylase (PEPC), phosphoenolpyruvate carboxykinase (PEPCK), and Ribulose 1,5-bisphosphate carboxylase-oxygenase (Rubisco) is the key enzyme of the C3 pathway [26], we measured the relative expression levels of the three genes in the conchocelis and thallus, respectively, of *P. haitanensis* by qRT-PCR. Results of the qRT-PCR (Figure 5) showed that the expression level of PEPC and PEPCK were 2.5-fold and 91.2-fold higher, respectively, in the conchocelis than in the thallus (*P*<0.01). However, the expression level of Rubisco in the conchocelis was 4.7-fold lower than in the thallus (*P*<0.01). The difference in expression levels of the three genes at different stages of the *P. haitanensis* life cycle indicated that the carbon fixation pathways were different. Therefore, the C4-like carbon fixation pathway may occur in the conchocelis stage of *P. haitanensis*.

**Table 2:** **Enzyme codes and corresponding gene names related to the carbon fixation pathway in *P. haitanensis***

**Enzyme codes**	**Enzyme names**	**Unigenes**
C3 pathway		49
EC 4.1.1.39	Ribulose bisphosphate carboxylase/oxygenase (Rubisco)	2
EC 2.7.2.3	Phosphoglycerate kinase (PGK)	3
EC 2.1.13/59	Glyceraldehyde-3-phosphate dehydrogenase (phosphorylating) (GAPDH)	1
EC 5.3.1.1	Triose-phosphate isomerase (TIM)	6
EC 4.1.2.13	Fructose-bisphosphate aldolase	17
EC 3.1.3.11	Fructose-1,6-bisphosphatase (FBPase)	10
EC 2.2.1.1	Transketolase	3
EC 3.1.3.37	Sedoheptulose-bisphosphatase (SBPase)	3
EC 5.3.1.6	Ribose-5-phosphate isomerase	1
EC 5.1.3.1	Ribulose-phosphate 3-epimerase	2
EC 2.7.1.19	Phosphoribulokinase	1
EC 4.1.2.9	Phosphoketolase	0
EC 4.1.2.22	Fructose-6-phosphate phosphoketolase	0
C4 pathway		24
EC 4.1.1.49	Phosphoenolpyruvate carboxykinase (PEPCK)	4
EC 4.1.1.31	Phosphoenolpyruvate carboxylase (PEPC)	1
EC 2.6.1.1	Aspartate aminotransferase (AST)	4
EC 1.1.1.37	Malate dehydrogenase (NAD +) (MD)	3
EC 1.1.1.39	Malate dehydrogenase (decarboxylating) (NAD +) (MD)	3
EC 2.7.1.40	Pyruvate kinase	5
EC 2.6.1.2	Alanine aminotransferase (ALT)	1
EC 1.1.1.40	Malic enzyme (ME)	3
EC 1.1.1.82	Malate dehydrogenase (NADP +)	0
EC 2.7.9.1	Pyruvate orthophosphate dikinase	0
Total		73

### Development and characterization of cDNA-derived SSR markers

For development of new molecular markers for *P. haitanensis*, all of the 24,575 unigenes generated in this study were used to mine potential microsatellites that were defined as dinucleotide to hexanucleotide SSRs with a minimum of four repetitions for all motifs. Using the MISA Perl script, http://pgrc.ipk-gatersleben.de/misa/, a total of 2,727 potential cSSRs were identified in 2,404 unigenes, of which, 261 sequences contained more than 1 cSSR, and 135 cSSRs were present in compound form (Table 3, Additional file 3). Mathematically, 9.78% *P. haitanensis*’ unigenes contain at least one SSR. Considering that approximately 15,859 kb were analyzed, we detected a frequency of at least one SSR per 5.8 kb in the expressed fraction of the *P. haitanensis* genome.

**Table 3:** **Summary of cSSR search results in *P. haitanensis***

**Search item**	**Number**
Total number of sequences examined	24,575
Total size of examined sequences (bp)	15,859,328
Total number of identified SSRs	2,727
Number of SSR containing sequences	2,404
Number of sequences containing more than 1 SSR	261
Number of SSRs present in a compound form	135

The 2,727 cSSRs contained four types of dinucleotide SSRs, nine types of trinucleotide SSRs, six types of tetranucleotide SSRs, nine types of pentanucleotide SSRs, and thirty types of hexanucleotide SSRs (Table 4, Additional file 4). Among all the cSSRs, trinucleotides were the most common type of SSR, accounting for 87.17%. The second most common type of SSR was dinucleotide, accounting for 8.87%. Tetranucleotides, pentanucleotides, and hexanucleotides were not common (Table 4).

**Table 4:** **Number and frequency of repeat types in the cSSRs of *P. haitanensis***

**Repeat types**	**Number of motif types**	**Number of cSSRs**	**Percentage of all SSRs (%)**	**Frequency in all unigenes (%)**
Dinucleotides	4	242	8.87	0.98
Trinucleotides	9	2377	87.17	9.67
Tetranucleotides	6	11	0.40	0.05
Pentanucleotides	9	17	0.62	0.07
Hexanucleotides	30	80	2.94	0.33
Total	58	2727	100	11.10

Of the dinucleotide repeats in the cSSRs of *P. haitanensis*, AC/GT was most common, accounting for 42.56%, and the most common number of repeats was 12 (Additional file 4). The most common trinucleotide repeat was CCG/GCC with 68.91% of all trinucleotide repeats found in *P. haitanensis* unigenes. This was followed by AGC/CTG with 11.44% and ACC/GGT with 6.86%. All other types of trinucleotide repeats combined constituted 13%, and AAT/TTA repeats were not found (Additional file 4).

Not all SSRs were suitable for primer design. Out of 2,727 cSSRs, primer-pairs could only be designed for 824. Detailed information on the designed primers is shown in Additional file 4. For the remaining 1,903 EST-SSRs, primer-pairs could not be designed for one of the following reasons: (1) SSRs were located too close to the end of the flanking region to accommodate primer design or (2) the base composition of the flanking sequence was unsuitable.

Among the 824 primer pairs, 100 primer pairs were randomly selected to evaluate their application across six wild strains of *P. haitanensis* (Additional file 5). Eighty-seven of the 100 primer pairs resulted in successful PCR amplification. The remaining 13 primers failed to generate PCR products at various annealing temperatures and Mg^2+^ concentrations and would be excluded from further analysis. Of the 87 working primer pairs, 62 amplified PCR products at the expected sizes, and 11 primer pairs resulted in larger PCR products than expected, suggesting there may be an intron within the amplicons. PCR products from the other 4 primer pairs were smaller than expected, suggesting a deletion might have occurred within the genomic sequence, lack of specificity of the primers, or an assembly error.

## Discussion

### *De novo* transcriptome sequencing and assembly of *P. haitanensis*

Transcriptome sequencing is one of the most important tools for expression pattern identification and gene discovery [15-21,27]. In the present study, to identify as many genes as possible, a cDNA library was constructed from mixed samples consisting of both gametophytes and sporophytes at different developmental stages and under different stress conditions. High-throughput sequencing technology was used to analyze the global transcriptome of *P. haitanensis*. Because of its great efficiency and accuracy, NGS technology has become a tremendous approach for high-throughput transcriptome analysis [18,21]. However, because of the relatively short read length, Illumina sequencing was mainly limited to organisms with available genomes as a reference [19,27]. Over the last few years, relatively short reads have been effectively assembled [19-21], especially with the advantage of paired-end sequencing [28]. Therefore, the Illumina transcriptome or whole genome *de novo* sequencing and assembly have been successfully used for non-model organisms [11,29-35]. Consistent with these publications, our results also indicated that relatively short reads from Illumina paired-end sequencing can be effectively assembled. Here, approximately 103 million 90 bp paired-end reads were generated from an Illumina HiSeq 2000. Based on paired-end information, these reads were assembled into 24,575 unigenes. The average length of these unigenes was 645 bp which was longer than those assembled in previous studies, such as the Whitefly (266 bp) [30], Oriental fruit fly (454 bp) [31], Sweet potato (581 bp) [32], peanut (619 bp) [33], rubber tree (485 bp) [34], banana (554 bp) [35] and *P. yezoensis* (419 bp) [11]. The assembled quality of these unigenes has been confirmed by RT-PCR amplification and Sanger sequencing for 10 randomly selected unigenes. The proportion of unigenes that match to known proteins is also an important index of assembly quality. In this study, all the unigenes were further subjected to BLASTx analysis against public databases, and 16,377 unigenes (66.64%) showed significant homology to functional genes encoding specific proteins. The unigenes encoding the majority of enzymes involved in the pathways of carbon fixation (including C3 and C4 pathways, Table 2) can be found in our database. These results indicate that relatively short reads from Illumina paired-end sequencing for a non-model organism can be effectively and accurately assembled.

Estimating the level of transcript coverage represented in a unigene collection is an important issue for transcriptome sequencing projects, but it is difficult or impossible without a completely annotated reference genome sequence [11,34]. Here, we indirectly evaluated transcriptome coverage by searching the ESTs downloaded from GenBank against unigenes from this study using BLASTn (E≤1.00×10^-7^). The comparison showed that 5,196 ESTs (86.10%) from GenBank matched 3,661 unigenes from this study (Additional file 6). This result indicated that the pool of transcript sequences covered a majority of the transcriptome of *P. haitanensis*.

From the transcriptome sequencing, we also found that the average GC-content in all the unigenes of *P. haitanensis* was 63.99%. This is significantly higher than in modern plants, such as *Arabidopsis thaliana* (41.10%), *Oryza sativa* (47.52%), *Adiantum capillus-veneris* (45.97%), *Physcomitrella patens* (47.60%), *Marchantia polymorpha* (54.75%) and *Chlamydomonas reinhardtii* (57.22%) [36], but is similar to *P. yezoensis* (63.2%) [11]. These results are in agreement with other genomic comparative analyses of a wide range of plant groups, where more primitive plant group genomes have a higher GC-content [37]. The high GC-content in unigenes obtained in the present study can be a result of codon usage preference.

These results indicate that high-throughput RNA-sequencing is an efficient, inexpensive, and reliable platform for transcriptomic analysis in non-model organisms. The large number of sequences generated in this study provides valuable sequence information for *P. haitanensis* at the transcriptomic level for marker development, novel gene discovery and for analyzing the gene expression profile under different physiological conditions. Although a large number of unigenes were obtained in this study, most of them were partial sequences of specific genes and some of the unigenes were allelic variants or located in different parts of the same gene. Because of short size or poor alignment, some sequences were excluded from analysis. When using this type of data to find genes of interest particular attention should be paid to identifying each unigene to confirm that it is unique. To solve this problem, RACE technology is the preferred choice for classification and obtaining the full length of these genes.

### Pathway of carbon fixation in *P. haitanensis*

Photosynthesis is one of the most important physiological processes in all green plants as well as algae, and carbon fixation is the most dominant step. The carbon fixation pathways in photosynthesis can be divided into three general categories: C3, C4 and Crassulacean acid metabolism (CAM) [38]. Among the three pathways, the C3 pathway, also known as the Calvin cycle, is the most basic and universal form of net carbon fixation based on Rubisco. The majority of plants, including many important crops, such as rice, wheat, soybean, and potato, assimilate CO_2_ using this pathway [38]. However, the C4 and CAM pathways are adjuncts of the C3 pathway that developed novel and efficient CO_2_ concentration mechanisms to enhance Rubisco performance even at limiting ambient CO_2_ levels [26]. For example, it has been shown that C4 plants can achieve at least 2-fold higher rates of carbon assimilation and biomass production over C3 plants [26]. In contrast, CAM plants show lower rates of net photosynthesis, but are capable of growing in conditions of even extreme aridity, such as deserts [38].

The physiological, biochemical, and molecular features of the C4 pathway have been extensively studied in higher plants for their potential in improving the productivity of crops [39,40]. The C4 pathway in algae has also been the subject of several reports in the last decade. Reinfelder et al. [41,42] first reported that the C4 pathway supported carbon assimilation in the marine diatom *Thalassiosira weissflogii*. Genomic data also provided evidence for the existence of key enzymes involved in the C4 metabolism in diatoms [43]. Roberts et al. [44] further reported that C3 (glycerate-P and triose-P) and C4 (mainly malate) compounds were all initial products in photosynthesis of *T. weissflogii* by short-term metabolic ^14^C labeling. In addition, some intermediate products of the C4 pathway had been detected in diatoms, brown alga, euglenoids, and dinoflagellates [45-49], and C4-like photosynthetic characteristics also had been detected in green algae [50].

In *Pyropia*, genes of the key enzymes in the C4 pathway, such as PEPCK and aspartate aminotransferase (AST) were identified in *P. haitanensis* sporophytes by analyzing the ESTs; however, PEPC was not detected [13]. By transcriptome sequencing nearly all of the key genes involved in the C4 pathway also have been identified in *P. yezoensis*[11]. In the present study, by transcriptome sequencing and gene annotation, genes of the key enzymes in the C3 and C4 pathways were also identified, including PEPC, which is the key enzyme responsible for CO_2_ assimilation in the first step of carbon fixation (Table 2). These results suggest that an efficient C4-like carbon fixation pathway might occur in *Pyropia*. As Luo et al. [24] reported, the inorganic carbon utilization in sporophytes and gametophytes of *P. haitanensis* is greatly different. We further measured the relative expression levels of PEPC, PEPCK (key genes in the C4 pathway) and Rubisco (key gene in the C3 pathway) in the conchocelis and thallus of *P. haitanensis* by qRT-PCR. The results showed that the expression level of Rubisco was significantly lower in the conchocelis than in the thallus, while the expression levels of PEPC and PEPCK were significantly higher in the conchocelis than in the thallus. The great difference in expression levels among the three genes in the different stages of the *P. haitanensis* life cycle further illustrated the different carbon fixation pathways in the conchocelis and thallus, and indicated that the C4-like pathway might play an important role in the fixing of inorganic carbon in the conchocelis stage of *P. haitanensis*.

### Development and characterization of cSSRs in *P. haitanensis*

SSRs have become important molecular markers for a broad range of applications. These include genome mapping and characterization, phenotype mapping, marker-assisted selection of crop plants and a range of molecular ecology and diversity studies [51]. However, few were used in *Pyropia* research because the standard methods to develop SSR-markers are time-consuming and expensive. Until now, only Zuo et al. [52] reported 11 polymorphic SSR loci obtained from *P. haitanensis* through an enriched genomic library.

With the development of high-throughput sequencing technology, a mass of sequence information has been incorporated into online databases. These allow us to develop SSR markers *in silico* efficiently and cost-effectively, and several crop plants have successfully developed large scale of SSR markers using this method [36]. In recent years, *in silico* development of SSR markers have also been performed in *Pyropia*. Liu et al. [53] first isolated 211 non-redundant SSR loci from 20,979 EST sequences of *P. yezoensis*, and 15 loci were selected for designing microsatellite primers. Sun et al. [54] mined 391 SSRs from 20,979 EST sequences of *P. yezoensis* with SSRIT software. From the mined SSRs, 48 SSR primer-pairs were designed and tested by commonly used SSR reaction conditions using 22 *Pyropia* DNA samples as templates. Wang et al. [55] discovered that 1,162 of 21,954 ESTs of *P. yezoensis* contained microsatellites. In *P. haitanensis*, Xie et al. [56] also identified 224 SSRs from 3,489 non-redundant *P. haitanensis* ESTs. From the 224 SSRs, 37 SSR primer-pairs were designed and tested using 15 *P. haitanensis* DNA samples. In this work, 2,727 SSRs loci, contained in 2,404 unigenes, were identified from 24,575 unigenes, and 824 of them were used to design high quality primers. One hundred primer pairs were randomly selected to evaluate their application in 6 wild strains of *P. haitanensis* and 87 successfully yielded amplicons. Among the successful primer pairs, 62 resulting amplicons were of the expected size. These results indicated that the assembled unigenes were of high quality and that most of the cSSR markers developed in this study could be used for a range of future studies in *P. haitanensis*.

SSRs are distributed in all regions of eukaryotic genomic DNA, both non-coding (such as introns or intergenic spaces) and coding regions [57]. Usually SSRs exist in 1–5% of EST sequences in plants [58]. In the present study, approximately 9.78% of all the *P. haitanensis* unigenes contained SSRs. This was more than what was found in *A. thaliana* (0.84%), *C. reinhardtii* (2.41%), *O. sativa* (3.57%), *M. polymorpha* (4.33%), *P. patens* (3.46%) [36] and *P. yezoensis* (3.4%) [11]. The reason for this is unclear, although it could be related to the small size of the *P. haitanensis* genome.

Like the statistical criterion of Cardele et al. [59], the highest frequency of the EST-derived SSRs was found in rice, at 3.4 kb between SSRs, followed by soybean (7.4 kb), maize (8.1 kb), tomato (11.1 kb), Arabidopsis (13.8 kb), poplar (14.0 kb) and cotton (20.0 kb). An overall average for these species was one SSR for every 5.4 kb (7,193 SSRs found in 38,502 kb of sequence) [59]. In *P. haitanensis*, the frequency of the unigene-derived SSRs was one SSR every 5.8 kb (2,727 SSRs found in 15859 kb of sequence), which is similar to the frequency previously observed in this species.

Although criteria for cSSR screening in different plants vary, the most common SSR motifs are trinucleotide repeats, which consist of 30% to 78% of plant SSR motifs [60]. In *P. haitanensis*, the result is in agreement with earlier studies. Of the 2,727 SSRs, 2,377 (87.17%) were trinucleotide repeats. Among all the trinucleotide repeat types, the GCC/CCG motif was the most common, accounting for 68.91% (1,638 of 2,377). The same results have also been found in the algae *P. yezoensis*[11], *C. reinhardtii* and the model moss *P. patens*[36], which might reflect the high GC-content in these species.

## Conclusions

In this study, high-throughput sequencing technology was first used to analyze the global transcriptome of *P. haitanensis* and 24,575 unigenes have been *de novo* assembled based on paired-end information. These unigene sequences constituted the first genomic resources for *P. haitanensis* and supplied some valuable resources for new gene discovery and cSSR marker development. Many genes generated in the present study will certainly accelerate the understanding of the molecular mechanisms of each specific trait of *P. haitanensis*, in particular for elucidating the complex mechanisms of stress tolerance in *P. haitanensis*. The discovery of the complete set of essential genes involved in the C3 and C4 carbon fixation pathways and a C4-like pathway may play important roles in the fixing of inorganic carbon in the conchocelis stage. This has helped us to clearly outline the panoptic view of carbon fixation in *P. haitanensis*. Additionally, in these generated sequences, 2,727 cSSRs were identified and characterized as potential molecular markers for *P. haitanensis.* These cSSR markers will enable genetic linkage map construction, gene-based association studies and marker assisted selection in *P. haitanensis* breeding. These results suggested that transcriptome analysis based on Illumina paired-end sequencing is a cost-effective and reliable approach to novel gene discovery and molecular marker development in a non-model organism.

## Methods

### Materials and cultivation conditions

The thallus (gametophytes) and free-living conchocelis (sporophytes) of *P. haitanensis* were cultured under different conditions (Additional file 1). The thallus was cultured in natural seawater, and the free-living conchocelis was cultured in natural seawater with Provasoli’s enrichment solution (PES) medium.

### Preparation of total RNA

Total RNA was isolated from each sample listed in Additional file 1. The collected samples were first cleaned with sterilized water. After drying with hygroscopic filter paper, the samples were ground into powder with liquid nitrogen. RNA was extracted and purified by E.Z.N.A.™ Plant RNA Kit (OMEGA, Germany). The quality and quantity of the purified RNA were determined by measuring the absorbance at 260 nm/280 nm (A260/A280) using a Nanodrop® ND-1000 spectrophotometer (LabTech, Holliston, MA, USA). RNA quality was further verified using a 2100 Bioanalyzer RNA Nanochip (Agilent, Santa Clara, CA), and all samples had RNA Integrity Number (RIN) values greater than 8.5. Finally, 5 μg RNA of each sample was pooled into one sample for cDNA library construction.

### Library construction and sequencing

The cDNA libraries were constructed following the manufacturer’s instructions (Illumina). Briefly, poly(A) RNA was isolated from 10 μg of total RNA using Oligo (dT) magnetic beads. Following purification, the mRNA was fragmented into small pieces and the cleaved RNA fragments were used for first stand cDNA synthesis using reverse transcriptase and random primers. This was followed by second-strand cDNA synthesis using buffer, dNTPs, RNaseH and DNA polymerase I. These cDNA fragments were then purified with a QiaQuick PCR extraction kit (Qiagen, Germany) and resolved with EB buffer for end reparation and poly(A) addition. The cDNA fragments were then connected with sequencing adapters. After agarose gel electrophoresis, the suitable fragments were selected as templates for PCR amplification to create the final cDNA library. The library was sequenced using Illumina HiSeq 2000 at Huada Genomics Institute Co. Ltd, China.

### Data filtering and *de novo* assembly

The quality requirement for *de novo* transcriptome sequencing is far higher than that for re-sequencing because sequencing errors can create difficulties for the short-read assembly algorithm. Therefore, before *de novo* assembly, raw reads produced from sequencing machines were cleaned by removing adaptors, empty reads, reads in which unknown bases were more than 5%, and low quality reads (where more than 10% of bases in a read had a quality value Q < 20).

Transcriptome *de novo* assembly was carried out with the short reads assembling program Trinity. Trinity (http://trinityrnaseq.sourceforge.net/) release 20110519 was used with the ALLPATHSLG error correction. The minimum contig length and paired fragment length were set to 100 bp and 180 bp, respectively [20]. Trinity first combined reads with a certain length of overlap to form contigs. Then all the cleaned reads were mapped back to the contigs. With paired-end reads it is possible to detect contigs from the same transcript as well as the distances between these contigs. Finally, Trinity connected the contigs and assembled sequences that could not be extended on either end. These sequences were defined as unigenes. The unigenes from each sample's assembly could be further processed by sequence splicing and removing redundancy with sequence clustering software to acquire non-redundant unigenes of the greatest length possible. In the final step, BLASTx alignment (E-value<0.00001) between unigenes and protein databases, such as Nr, Swiss-Prot, KEGG and COG was performed, and the best aligning results were used to decide the sequence direction of the unigenes. If results of different databases conflicted, then the Nr database had priority in determining sequence direction, followed by Swiss-Prot, then KEGG and finally COG. When a unigene was not aligned using the above databases, ESTScan software [61] was used to decide its sequence direction.

Read data are available from the Sequence Read Archive (SRA), accessible through NCBI BioProject ID PRJNA181961. Assembled contig sequences are deposited in the Transcriptome Shotgun Assemblies (TSA) database, which is accessible through NCBI BioProject ID GADD00000000.

### Unigene functional annotation

Annotation can provide information on the expression and function of unigenes. Unigene sequences were first aligned by BLASTx to the GenBank Nr and the Swiss-Prot protein databases with an E-value cutoff of 10^-5^. To estimate the number of annotated unigenes that matched to unique genes in the two databases, these files were then filtered for the duplicates in protein accessions. With the Nr annotation, the Blast2GO program [22] was used to get GO annotations according to molecular function, biological process and cellular component ontologies (http://www.geneontology.org). The unigene sequences were also aligned to the COG database to predict and classify possible functions. Pathway assignments were also carried out according to the KEGG pathway database [62] using BLASTx with an E-value threshold of 10^-5^.

### Gene expression in different stages of the *P. haitanensis* life cycle

The relative expression levels of PEPC, PEPCK, and Rubisco genes in the conchocelis and thallus stages were measured by Real-time fluorescent quantitative PCR (qRT-PCR) (Additional file 7). Total RNA was extracted and purified by E.Z.N.A.™ Plant RNA Kit (OMEGA, Germany). cDNA was synthesized with oligo (dT) and random hexamer primers by PrimeScript^R^ RT reagent kit (TaKaRa, Japan).

The qRT-PCR was performed with an ABI 7300 Real-time PCR Detection system and performed in a total volume of 20 μL containing 10 μL of 2×SYBR green Master Mix (ToYoBo, Japan), 2.0 μL (2 mM) of each primer, 2 μL of the diluted cDNA mix, and 4 μL of RNA-free water. The beta-tubulin (*TubB*) gene was used as an internal control gene [63]. The sequence of each primer is shown in Table 2. The thermal profile for qRT-PCR was 95°C for 1 min, followed by 40 cycles of 95°C for 10s, and 60°C for 30s. Dissociation curve analysis of the amplicons was performed at the end of each PCR reaction to confirm that only one specific PCR product was amplified and detected. qRT-PCR was performed in triplicate for each sample. After the PCR program, the data were analyzed with the ABI optical system software. To maintain consistency, the baseline was set automatically by the software. All data were given as mean ± SE in terms of relative mRNA expression. The results were analyzed with Student’s *t*-test, and P<0.05 was set as the level of statistical significance.

### Development of cDNA-derived SSR (cSSR) markers

A Perl script known as MIcroSAtellite (MISA, http://pgrc.ipk-gatersleben.de/misa/) was used to identify microsatellites in the unigenes. The parameters for the SSR search were defined as follows: the size of motifs was two to six nucleotides, and the minimum repeat unit was defined as six for dinucleotides, five for trinucleotides and tetranucleotides, and four for pentanucleotides and hexanucleotides. The frequency of cSSRs refers to kilobase pairs of cDNA sequence containing one SSR. Primer Premier 5.0 (PREMIER Biosoft International, Palo Alto, CA) was used to design PCR primers in the flanking regions of the SSRs. The criteria of the primer design were as follows: primer length of 18–24 bp; GC content between 40–65%; and melting temperature between 50–65°C. The expected product size was between 100 bp and 350 bp with no secondary structures. Among all the designed primers, 100 primer pairs were randomly selected to evaluate their application and polymorphisms in six wild strains of *P. haitanensis*.

## Abbreviations

COG: Clusters of orthologous groups; cSSR: cDNA simple sequence repeat; EST: Expressed sequence tag; GO: Gene ontology; KEGG: Kyoto Encyclopedia of Genes and Genomes; PEPC: Phosphoenolpyruvate carboxylase; PEPCK: Phosphoenolpyruvate carboxykinase; qRT-PCR: Real-time fluorescent quantitative PCR; Rubisco: Ribulose 1,5-bisphosphate carboxylase-oxygenase.

## Competing interests

The authors declare that they have no competing interests.

## Authors' contributions

CX and CC conceived and designed the experiments. CX and BL performed the experiments and data analysis. CC, YX and DJ helped to prepare the reagents and materials. CX and CC wrote the manuscript. All authors have read and approved the manuscript.

## Supplementary Material

Additional file 1 **Different samples of *P. haitanensis *for cDNA library construction.** Click here for file

Additional file 2 **Functional annotation of 24,575 unigenes identified in the transcriptome of *P. haitanensis*.** Click here for file

Additional file 3 **Identified cSSRs in unigenes of *P. haitanensis*.** Click here for file

Additional file 4 **Summary of microsatellite sequences identified from the unigenes of *P. haitanensis*.** Click here for file

Additional file 5 **Detailed information on the designed primers for 824 cSSRs in *P. haitanensis*.** Click here for file

Additional file 6 **Comparison of the identical sequences in *P. haitanensis *unigenes from this study with ESTs obtained from GenBank.** Click here for file

Additional file 7 **Information of the primers used in the qRT-PCR analysis of *P. haitanensis *genes.** Click here for file
