# Supplementary material for: Characterization of the global transcriptome for Pyropia haitanensis (Bangiales, Rhodophyta) and development of cSSR markers
Source: BMC Genomics. 2013 Feb 16;14:107. doi: 10.1186/1471-2164-14-107 (PMC3626662; doi:10.1186/1471-2164-14-107)
Supplement: Additional file 1 — Different samples of P. haitanensis for cDNA library construction. [file 1471-2164-14-107-S1.docx]

**Additional file 1: Different samples of *P. haitanensis* for cDNA library construction**

| Samples | Cultivation conditions |
| --- | --- |
| Free-living conchocelis of Z-61 strain^1^ | 21°C, 50 µmol photons m^−2^ s^−1^ (12L:12D) |
| Free-living conchocelis of wild-type strain ^2^ | 21°C, 50 µmol photons m^−2^ s^−1^ (12L:12D) |
| Free-living conchocelis of Pigment mutant (Breen)^3^ | 21°C, 50 µmol photons m^−2^ s^−1^ (12L:12D) |
| Thallus of Z-61 strain | 21°C, 50 µmol photons m^−2^ s^−1^ (12L:12D) |
| Thallus of Z-61 strain | 29°C, 50 µmol photons m^−2^ s^−1^, 6h |
| Thallus of Z-61 strain | 21°C, 1500 µmol photons m^−2^ s^−1^, 3h |
| Thallus of Z-61 strain | Drought conditions in controlled room for 3h, 21°C , water content at 25% |
| Thallus of wild-type strain | 21°C, 50 µmol photons m^−2^ s^−1^ (12L:12D) |
| Thallus of Pigment mutant (Breen) | 21°C, 50 µmol photons m^−2^ s^−1^ (12L:12D) |

Note: ^1^The Z-61 strain, which has the characters of high-temperature tolerance and high yield, was selected and purified by the Laboratory of Germplasm Improvements and Applications at Jimei University, Fujian Province, China.

^2^ The wild-type strain was established in 1999 from a gametophytic blade collected on the coast of Dongshan Island, Fujian Province, China, and has been maintained in the Laboratory of Germplasm Improvements and Applications at Jimei University, Fujian Province, China.

^3^ The pigment mutant of Breen was selected and purified by the Laboratory of Germplasm Improvements and Applications at Jimei University, Fujian Province, China.
