# Supplementary material for: Characterization of the global transcriptome for Pyropia haitanensis (Bangiales, Rhodophyta) and development of cSSR markers
Source: BMC Genomics. 2013 Feb 16;14:107. doi: 10.1186/1471-2164-14-107 (PMC3626662; doi:10.1186/1471-2164-14-107)
Supplement: Additional file 4 — Summary of microsatellite sequences identified from the unigenes of P. haitanensis. [file 1471-2164-14-107-S4.docx]

**Additional file4: Summary of microsatellite sequences identified from the unigenes of *P.haitanensis***

| Repeat types | Repeat motif | Count of cSSRs | Highest number of repeats |
| --- | --- | --- | --- |
| Dinucleotides | AC/GT | 103 | 12 |
|  | AG/CT | 37 | 11 |
|  | AT/AT | 4 | 6 |
|  | CG/CG | 98 | 12 |
| Trinucleotides | AAC/GTT | 20 | 30 |
|  | AAG/CTT | 25 | 24 |
|  | ACC/GGT | 163 | 20 |
|  | ACG/CGT | 148 | 22 |
|  | ACT/AGT | 1 | 5 |
|  | AGC/CTG | 272 | 30 |
|  | AGG/CCT | 105 | 16 |
|  | ATC/ATG | 5 | 6 |
|  | CCG/CGG | 1638 | 18 |
| Tetranucleotides | AAGG/CCTT | 1 | 5 |
|  | ACGC/CGTG | 2 | 6 |
|  | AGCC/CTGG | 4 | 6 |
|  | AGGC/CCTG | 2 | 5 |
|  | CCCG/CGGG | 1 | 5 |
|  | CCGG/CCGG | 1 | 5 |
| Pentanucleotides | AAAAG/CTTTT | 2 | 5 |
|  | AAGGC/CCTTG | 1 | 4 |
|  | ACAGC/CTGTG | 1 | 4 |
|  | ACCCC/GGGGT | 1 | 4 |
|  | ACCGC/CGGTG | 2 | 4 |
|  | ACGGG/CCCGT | 4 | 5 |
|  | AGAGG/CCTCT | 1 | 4 |
|  | AGGGG/CCCCT | 4 | 5 |
|  | CCGCG/CGCGG | 1 | 4 |
| Hexanucleotides | AAAGGC/CCTTTG | 1 | 4 |
|  | AACAGC/CTGTTG | 2 | 6 |
|  | AACGAC/CGTTGT | 1 | 4 |
|  | AAGGAG/CCTTCT | 1 | 4 |
|  | AAGGCC/CCTTGG | 1 | 4 |
|  | ACACGC/CGTGTG | 1 | 4 |
|  | ACAGCG/CGCTGT | 2 | 4 |
|  | ACCACG/CGTGGT | 1 | 4 |
|  | ACCCGC/CGGGTG | 1 | 9 |
|  | ACCGCC/CGGTGG | 10 | 7 |
|  | ACCGCG/CGCGGT | 6 | 7 |
|  | ACCGGG/CCCGGT | 2 | 4 |
|  | ACGAGG/CCTCGT | 1 | 4 |
|  | ACGATG/ATCGTC | 1 | 12 |
|  | ACGCCC/CGTGGG | 1 | 4 |
|  | ACGCCG/CGGCGT | 6 | 4 |
|  | ACGCGG/CCGCGT | 1 | 4 |
|  | ACGGCC/CCGTGG | 2 | 6 |
|  | ACGGCG/CCGTCG | 7 | 4 |
|  | ACGGGC/CCCGTG | 1 | 12 |
|  | ACGTCC/ACGTGG | 1 | 4 |
|  | AGCCGC/CGGCTG | 3 | 10 |
|  | AGCCTG/AGGCTC | 1 | 4 |
|  | AGCGGC/CCGCTG | 11 | 8 |
|  | AGCGGG/CCCGCT | 8 | 4 |
|  | AGGCGC/CCTGCG | 2 | 9 |
|  | AGGCGG/CCGCCT | 1 | 4 |
|  | AGGGGC/CCCCTG | 1 | 4 |
|  | CCGCGG/CCGCGG | 1 | 4 |
|  | CCGGCG/CCGGCG | 2 | 4 |
