# Supplementary figures and images for: Characterization of the global transcriptome for Pyropia haitanensis (Bangiales, Rhodophyta) and development of cSSR markers
Source: BMC Genomics. 2013 Feb 16;14:107. doi: 10.1186/1471-2164-14-107 (PMC3626662; doi:10.1186/1471-2164-14-107)

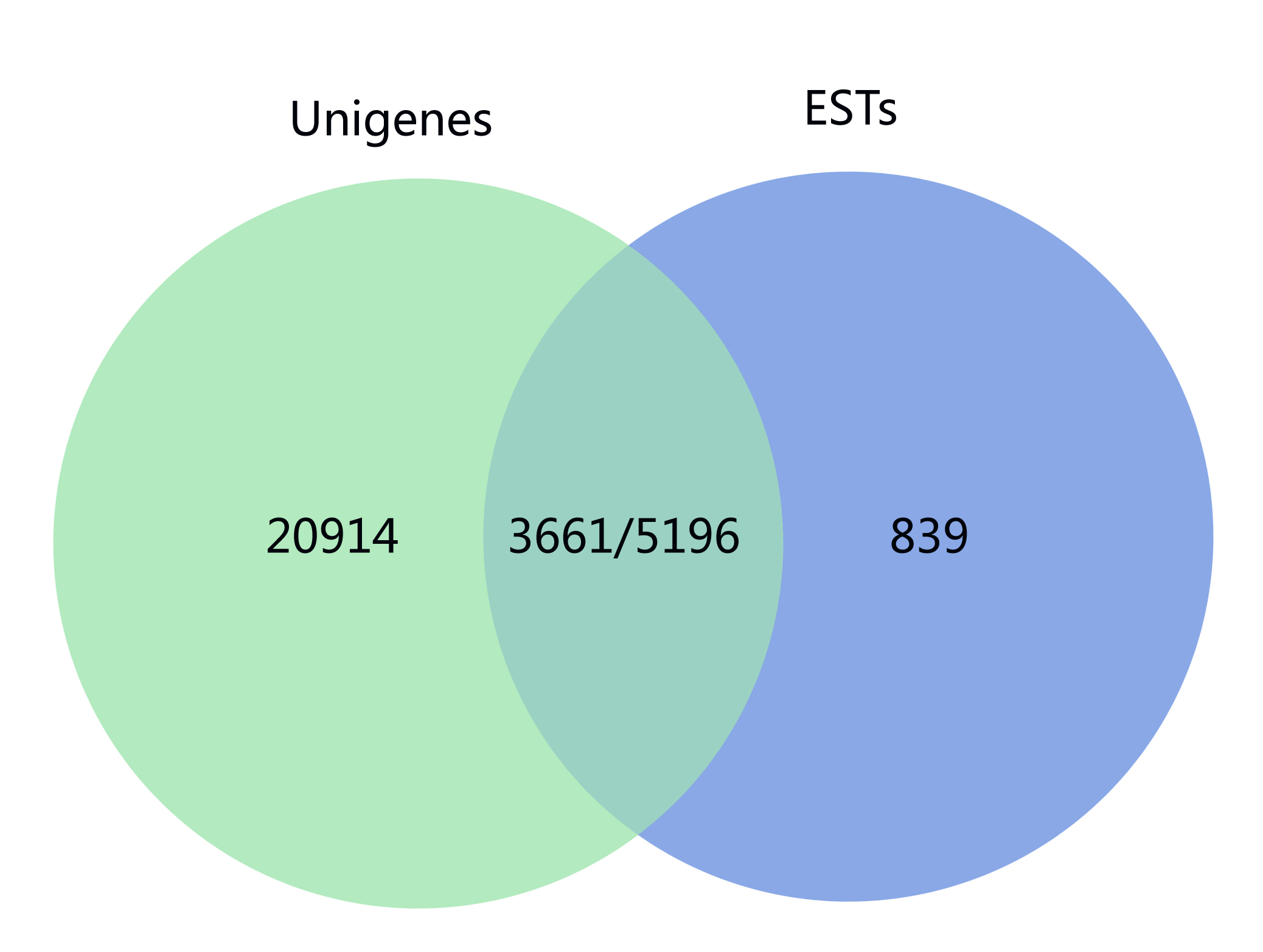

Supplement: Additional file 6 — Comparison of the identical sequences in P. haitanensis unigenes from this study with ESTs obtained from GenBank. [file 1471-2164-14-107-S6.jpeg]
