# Supplementary material for: Characterization of the global transcriptome for Pyropia haitanensis (Bangiales, Rhodophyta) and development of cSSR markers
Source: BMC Genomics. 2013 Feb 16;14:107. doi: 10.1186/1471-2164-14-107 (PMC3626662; doi:10.1186/1471-2164-14-107)
Supplement: Additional file 7 — Information of the primers used in the qRT-PCR analysis of P. haitanensis genes. [file 1471-2164-14-107-S7.docx]

**Additional file 7: Information of the primers used in the qRT-PCR analysis of *P. haitanensis* genes.**

| Gene name | Primer name | Sequence (5’-3’) | Product size (bp) |
| --- | --- | --- | --- |
| PEPC | PF | CAACCTGTCTGAGGAGGAACTGC | 163 |
|  | PR | ACGCAACACCTCGTCCACC |  |
| PEPCK | PKF | TCGGCGTTTATCACCTTTGACT | 162 |
|  | PKR | GCCATTCTCGTTGGTCGTG |  |
| Rubisco | RF | TTACTTAGGCGATGATGTAGTTCTT | 126 |
|  | RR | CCTTCATTTCTTGCCATAACC |  |
| *TubB* | TF | TGCAGGGCTTCCAGGTGAC | 207 |
|  | TR | CCGCATCCGCATTCTCCAC |  |
